# Effect of ElectroMagnetic interference from SmartPHone on cardiac ImplaNtable electronic device (EMI‐PHONE study)

**DOI:** 10.1002/joa3.12754

**Published:** 2022-07-12

**Authors:** Sanatcha Apakuppakul, Nilubon Methachittiphan, Sirin Apiyasawat

**Affiliations:** ^1^ Ramathibodi hospital Mahidol University Bangkok Thailand

**Keywords:** cardiac implantable electronic device, cardiac resynchronization therapy, electromagnetic interference, implantable cardioverterdefibrillator, pacemaker, smartphone

## Abstract

**Background:**

Smartphone can emit two types of electromagnetic waves, static field and dynamic field. Evidence showed the interference from phones to cardiac implantable electronic devices (CIEDs). The smartphones and CIEDs are reportedly better designed to reduce electromagnetic interference (EMI).

**Methods:**

80 consecutive subjects with CIEDs were recruited and tested for EMI. Subject was tested with three different smartphones (Nokia 3310, Iphone 7, and Samsung 9S). Phone was attached to chest wall at 0 cm at generator site, at atrial lead level, and at ventricular lead level. During the tests, real‐time interrogations were performed to detect any EMI from smartphone in standby mode, and during calling‐in and out for 30 s. After the tests, post‐test interrogations were performed to detect any parameter change. Adverse events including pacemaker inhibition, false ICD shock, CIEDs device malfunction, and urgent electrophysiologist consultations were recorded.

**Results:**

80 subjects (mean age 70.5‐year‐old, 50% male) recruited in the study, all completed the testing protocol. The most common type of CIEDs tested was pacemaker (*N* = 56, 70%), followed by ICD (*N* = 16, 20%), and CRT (*N* = 8, 10%). Most patients (*N* = 62, 77.5%) had more than one lead implanted. The mean year of implantation was 5.2±2.8 (devices were implanted from 2008 to 2019). Of all the tests performed, there was no EMI or adverse events observed.

**Conclusion:**

Current generation of smartphones has no EMI effect on CIEDs in our study and can be used safely with less concern about adverse events including pacemaker inhibition, inappropriate ICD shock, and CIEDs device malfunction.

## Graphical abstract

Current generation of smartphones have no EMI effect to CIEDs in our study and can be used safely with less concern about adverse events including pacemaker inhibition, inappropriate ICD shock and CIEDs device malfunction.

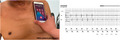

## 1 INTRODUCTION

Nowadays, the number of users and technology of smartphones growing rapidly. From the national database of Thailand in 2018, 51 million smartphones were registered.

Smartphones can emit two types of electromagnetic waves, static field from magnet and dynamic field from calling. Previous evidence showed the interference effects from generations of mobile phones to cardiac implantable electronic devices (CIEDs).^1^, ^2^

Theoretically, electromagnetic interference (EMI) from smartphones can affect cardiac implantable electronic devices (CIEDs) function by causing abnormal signals that result in CIEDs oversensing, leading to pacing inhibition of pacemaker and inappropriate shock in implantable cardioverter‐defibrillator (ICD).^3^, ^4^

There is a recommendation from device companies and the United States of America food and drug administration (US FDA) that mobile phones should be used at least 15 centimeters from CIEDs.^5^

However, this recommendation was derived from older generations of mobile phones and an older generation of cardiac implantable electronic devices (CIEDs). The current generations of smartphones and CIEDs are reportedly better designed to reduce the effects of electromagnetic interference (EMI). The modern smartphone technology utilizing 4G or 5G frequency spectrum emits less electromagnetic waves than that of 2G or 3G technology.

There were a few studies that showed the safety of using smartphones less than 15 centimeters distance, but the number of subjects was small^6^ and only a few models of mobile phones were tested.^7^

We seek to find the presence and the magnitude of EMI from the current generation of smartphones and their effects on current models of CIEDs.

## 2 METHODS

### 2.1 Study population

Patients with cardiac implantable electronic devices (CIEDs) were recruited from the Ramathibodi device clinic. Patients aged between 18 and 80 years old with any type of CIEDs were included in our study. Exclusion criteria are any abnormal CIEDs parameter during regular scheduled device interrogation, for example, abnormal threshold, abnormal impedance, any leads, and pulse generator issues. Pacemaker‐dependent patients were excluded for safety reasons. During test protocol, patient was closely monitored by cardiology fellow and device technician. Informed consent was obtained from every patient.

We initially planned to recruit at least 76 subjects in order to detect endpoints using 27% for the effect of electromagnetic interference from previous study^3^ and 10% different from expected event in our study. Finally, we recruited 80 subjects from September 2018 to December 2019.

After patients visited the Ramathibodi device clinic on a regular follow‐up schedule and devices parameters were in good range, the patients were asked to give informed consent and follow protocol testing. Baseline characteristics including age, gender, functional class, device type, mode of device, indication of implantation, position of implantation, number of leads, duration of implantation, LVEF, and underlying heart disease were recorded.

**FIGURE 1:**
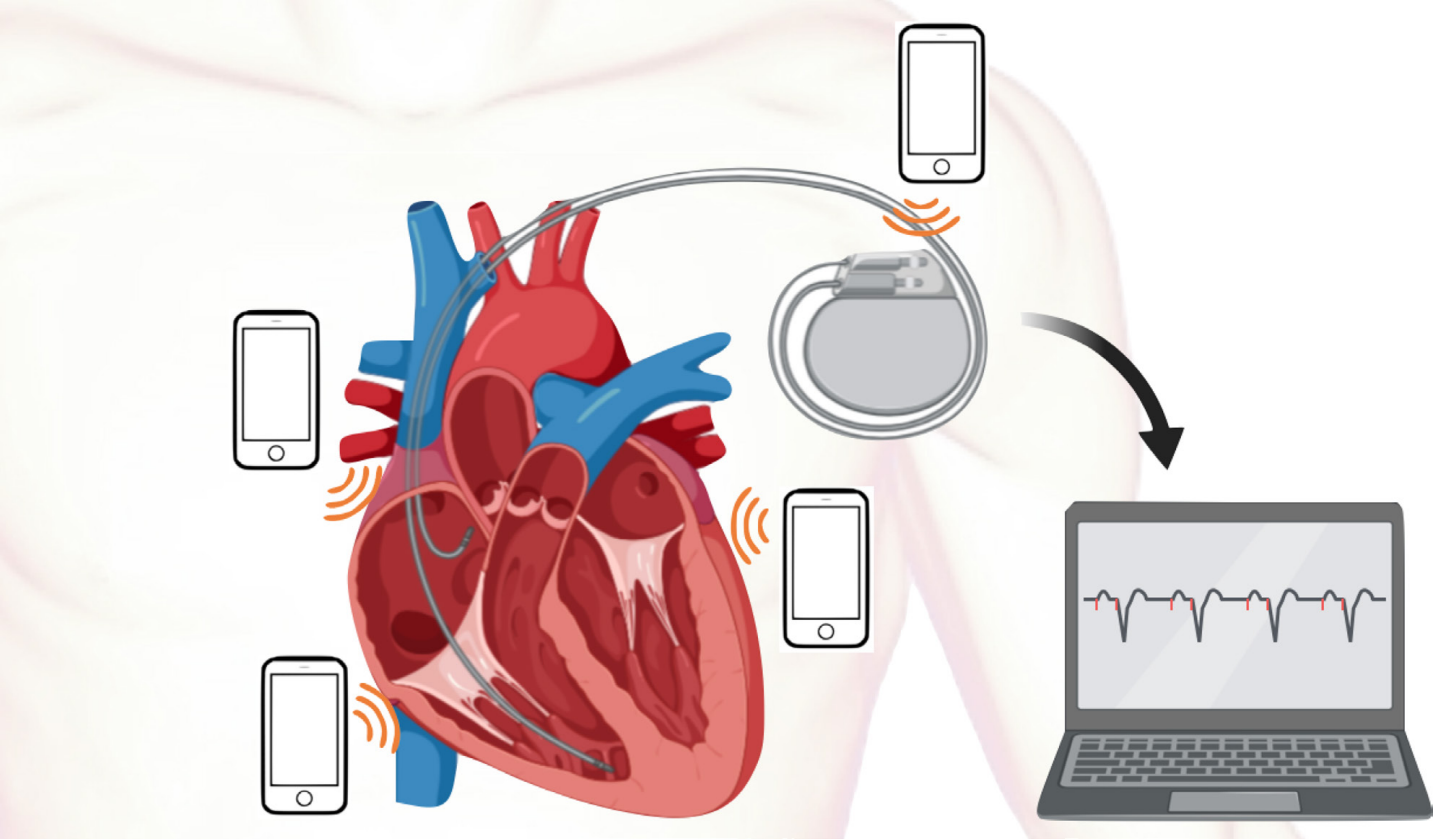
Infographic of testing protocol.

**FIGURE 2:**
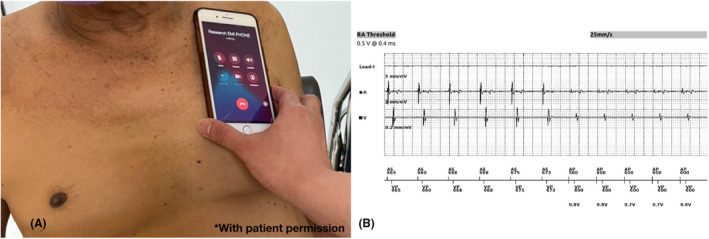
Real case illustration: (A) during testing of EMI from smartphone by calling out with attach directly to pacemaker. (B) Real time device interrogation in the patient using wireless device interrogation, the tracing revealed no EMI was detected and no negative effect to pacemaker. *with patient permission.

Smartphones were applied at 0 centimeter distance from patient's chest wall. The position of smartphones was changed every 30 s during the test. These positions included pulse generator position, right parasternal border (right atrial lead site, if present), left parasternal border (right ventricular lead site), and apical area (left ventricular lead site, if present). Smartphones were tested in standby mode, 30‐s calling‐in, and calling‐out. Smartphone brands used in our study are Nokia 3310, Iphone 7, and Samsung 9S. Each patient was tested with all three models in all three modes (i.e., Standby, calling‐in, and calling‐out) with each phone placed at different positions as stated above. During the test protocol, real‐time interrogation of device was done for detection of electromagnetic interference (EMI), pacemaker inhibition, and inappropriate ICD shock. Patients were closely monitored by cardiology fellow and device technician. All real‐time interrogated signals were recorded. After protocol testing, device interrogation was done in all subjects to detect any parameter changes from baseline. The testing protocol infographic was illustrated in Figure 1. Real‐case illustration in Figure 2.

### 2.2 Statistical analysis

Baseline characteristics are described as mean ± SD for continuous data and proportions for categorical data. All CIEDs parameters are analyzed for detection of difference between pre‐ and post‐test protocol, using t‐test analysis. *p*‐value less than .05 was used to define statistical significance. We use SPSS version 23 for data analysis.

### 2.3 Ethical issues

All patient identifications were concealed prior to analysis, using number of case record form. The ethic approval for data collection and analysis were approved by the institutional review boards committees of Mahidol university. This study complied with international guidelines for human research protection, Declaration of Helsinki, the Belmont report, CIOMS guidelines, and the international conference on harmonization in good clinical practice (ICH‐GCP). This study was funded by the Ramathibodi research foundation after approval by ethic committee.

### 2.4 Outcomes measurement

In our study, primary outcome was electromagnetic interference detected by real‐time device interrogation. Every intracardiac electrogram was adjudicated by a device technician. In case of any suspicion of EMI, adjudication with an electrophysiology fellow was performed. Secondary outcomes are device malfunction detected post‐protocol interrogation, pacing inhibition, inappropriate ICD shock, urgent electrophysiologist consultation, and CCU (cardiac care unit) admission.

## 3 RESULTS

### 3.1 Baseline characteristics

A total of 240 tests were performed on 80 enrolled subjects. Of all 80 subjects enrolled in our study, 40 (50%) patients were male. Mean age was 70.5 years old. NYHA functional class I and II were 92.5% and 7.5%, respectively. Most of the CIEDs devices were pacemaker (56 subjects, 70%), and the others were ICD (16 subjects, 20%), CRT‐P (3, 3.8%), and CRT‐D (5, 6.3%). Mode of devices were DDD (52 subjects, 65%), VVI (20 subjects, 25%), and biventricular pacing (8 subjects, 10%). The majority of implanted device position was left pectoral region (74, 92.5%) subjects. Patient who had 2‐lead device were 53 subjects (66.3%), 1‐lead were 18 (22.5%) subjects, and 3‐lead were 9 (11.3%) subjects. Mean duration of implantation was 5.2 ± 2.8 years. Mean LVEF was 56.4 ± 16.9%. Indication of implantation and underlying cardiac disease were described in Table 1. Models of devices are described in Table 2.

**TABLE 1:** Baseline characteristics

Baseline characteristics	*N* (%)
Age (Mean ± SD)	70.5 ± 12.9 year‐old
Gender (Male)	40 (50%)
Functional class (NYHA)	
NYHA class I	74 (92.5%)
NYHA class II	6 (7.5%)
Device type	
Pacemaker	56 (70%)
ICD	16 (20%)
CRT‐P	3 (3.8%)
CRT‐D	5 (6.3%)
Mode of device	
VVI (R)	20 (25%)
DDD (R)	52 (65%)
BiV	8 (10%)
Left pectoral implantation	74 (92.5%)
Number of leads	
1 lead	18 (22.5%)
2 leads	53 (66.3%)
3 leads	9 (11.3%)
Duration of implantation	5.2 ± 2.8 years
Years of implantation	
2008–2010	8 (10%)
2011–2013	20 (25%)
2014–2016	24 (30%)
2017–2019	28 (35%)
LVEF (Mean ± SD)	56.4 ± 16.9%
LVEF≤40%	18 (22.7%)
Underlying cardiac disease	
Sinus node dysfunction	21 (26.5%)
Coronary artery disease	16 (20%)
Valvular heart disease	
Severe AS	3 (3.9%)
Severe MR	4 (5.1%)
Severe PR	1 (1.3%)
Severe TR	1 (1.3%)
Cardiomyopathy	
DCM	14 (17.5%)
ICM	11 (13.8%)
HCM	3 (3.8%)
ARVC	2 (2.5%)
Congenital heart disease	
ASD	1 (1.3%)
TOF	1 (1.3%)
Indication of device implantation	
Sinus node dysfunction	25 (31.5%)
AV block	33 (41%)
SCD: Primary prevention	14 (17.5%)
SCD: Secondary prevention	8 (10%)

Abbreviations: ARVC: Arrhythmogenic right ventricular cardiomyopathy, AS: Aortic valve stenosis, ASD: Atrial septal defect, AV: Atrioventricular, CRT‐D: Cardiac resynchronization therapy – Defibrillator, CRT‐P: Cardiac resynchronization therapy – Pacemaker, DCM: Dilated cardiomyopathy, HCM: Hypertrophic cardiomyopathy, ICD: Implantable cardioverter‐defibrillator, ICM: Ischemic cardiomyopathy, LVEF: Left ventricular ejection fraction, MR: Mitral valve regurgitation, NYHA: New York Heart Association, PR: Pulmonic valve regurgitation, SCD: Sudden cardiac death, TOF: Tetralogy of Fallot, TR: Tricuspid valve regurgitation.

**TABLE 2:** Device model

Device model	*N* (%)
Boston scientific	
INOGEN MINI (D002)	1 (1.3%)
INOGEN EL (D140, D141)	7 (8.8%)
TELIGEN (F102, F103)	2 (2.5%)
AUTOGEN (F140, G058)	4 (5%)
LATITUDE (F141, F143)	2 (2.5%)
RESONATE (G447)	1 (1.3%)
CONTAK RENEWAL TR2 (H145)	1 (1.3%)
INGENIO (J063, J065, J066, J177)	10 (12.5%)
ALTRUA (S501, S502, S503)	9 (11.3%)
ESSENTIO (L110, L111)	22 (27.5%)
VISIONIST (U228)	1 (1.3%)
Medtronic	
ADAPTA DR (ADDR01)	1 (1.3%)
CONSULTA CRT‐P (C3TR01)	1 (1.3%)
VIVA S CRT‐D (DTBB2D1)	2 (2.5%)
EVERA MRI S (DVMC3D1, DDMC3D4)	2 (2.5%)
ENSURA MRI (EN1DR01)	6 (7.5%)
SENSIA D (SED01, SEDR01)	4 (5%)
VERSA DR (VEDR01)	4 (5.0%)

### 3.2 Outcomes

A total of 240 tests were performed on 80 subjects. There was no electromagnetic interference, pacing inhibition, or inappropriate ICD shock detected. These results were consistent with the mobile phone devices being on standby mode, 30‐s calling‐in, and 30‐s calling out at any position and any brand of smartphones. There was no urgent EP (electrophysiologist) consultation or CCU (cardiac care unit) admission in our study. Event rate was described in Table 3. Upon repeated CIED interrogation, device parameters including pacing function, sensing function, impedance, and threshold, did not significantly change. Device parameter was described in Table 4.

**TABLE 3:** Event rate

Outcomes	Event (%)
Primary outcome	
Electromagnetic interference detection	0
Secondary outcomes:	
Pacing inhibition	0
False ICD shock	0
Urgent electrophysiologist consultation	0
Significant parameter change	0
CCU admission	0

**TABLE 4:** Device parameter, pre‐, and post‐test protocol

Parameter	Pre‐test (mean ± SD)	Post‐test (mean ± SD)	*p*‐value
RA pace	21.25 ± 30%	22.5 ± 30.6%	.79
RA sense	2.4 ± 2.1 mV	2.4 ± 2.2 mV	1.0
RA impedance	353 ± 220 Ω	359 ± 221 Ω	.86
RA threshold	0.46 ± 0.4 mV	0.45 ± 0.4 mV	.88
RV pace	56 ± 46%	56.1 ± 46.1%	.99
RV sense	8.2 ± 7.4 mV	8.6 ± 7.8 mV	.74
RV impedance	490 ± 101 Ω	480.1 ± 100 Ω	.53
RV threshold	0.9 ± 0.3 mV	0.9 ± 0.4 mV	1.0
LV pace	97.8 ± 2.9%	97 ± 3.5%	.74
LV sense	16.4 ± 6.5 mV	17.2 ± 7.1 mV	.89
LV impedance	779 ± 328 Ω	803 ± 280 Ω	.88
LV threshold	1.6 ± 1.1 mV	1.58 ± 0.98 mV	.97
Shock impedance	49.3 ± 10.2 Ω	50 ± 10 Ω	.83

Abbreviations: RA: Right atrium, RV: Right ventricle, LV: Left ventricle.

## 4 DISCUSSION

The presence of electromagnetic interference (EMI) from smartphones can affect cardiac implantable electronic devices (CIEDs) function by emission of noise. CIEDs can oversense the noises, leading to pacing inhibition in pacemaker and inappropriate shock in implantable cardioverter‐defibrillator (ICD). Werner I. et al. described electromagnetic interference of pacemakers by mobile phones.^3^ Chiladakis et al. described in‐vivo testing of digital cellular telephones in patients with implantable cardioverter‐defibrillators.^4^ However, in contemporary era, CIEDs and smartphones technologies have improved tremoendously. CIEDs had many new features, including noise detection mode. Smartphones were also improved in network signaling, including 3G, 4G, and 5G. Burri H et al. reported low risk of electromagnetic interference between smartphones and contemporary implantable cardioverter defibrillators.^6^ Lennerz C et al. reported safety of using smartphones on CIEDs with regard to EMI.^7^

In our study, we did not test the interaction between Wi‐Fi and electromagnetic interference. However, previous data revealed there was no EMI effect of Wi‐Fi devices on pacemaker, Wi‐Fi devices do not pose the risk of EMI to implantable pacemaker. The immunity level of pacemaker is much higher than the transmitting power of radiofrequency devices, even when exposed to field levels five times higher than allowed by current international regulation, and do not exhibit any degradation of their performances.^8^

In our study, we established the safety of using current generation smartphones and CIEDs. We revealed that no EMI was detected by real‐time interrogation of CIEDs in our test protocol. Moreover, device parameters were not affected either.

From this study, we demonstrated safety and extremely low‐risk effect of EMI on CIEDs. Current generation of smartphone can be used safely with less concern about adverse events including pacemaker inhibition, inappropriate ICD shock, and CIEDs device malfunction.

## 5 LIMITATIONS

First, we do not have a comparison group. Second, we have no electromagnetic machine to quantify the electromagnetic waves emitted by the mobile phones. Third, we have only two device companies (Boston Scientific, Medtronic), three mobile phone model (Nokia 3310, Iphone 7, and Samsung 9S), and 3G, 4G systems were tested in our study.

## 6 CONCLUSION

Current generation of smartphones has no EMI effect on CIEDs and can be used safely without any adverse events including pacemaker inhibition, inappropriate ICD shock, or CIEDs device malfunction. In our study, we support that current generations of smartphones can be used safely in patients with CIEDs with less concern for EMI.

## FUNDING INFORMATION

Ramathibodi research foundation.

## CONFLICT OF INTEREST

None.

## ETHICS STATEMENT

This study had been approved by institutional review board committee of Mahidol university.

## PATIENT CONSENT

All patients included in this study had given consent to participate.

## CLINICAL TRIAL REGISTRATION

None.
